# To Our Patrons—Greeting

**Published:** 1892-07

**Authors:** 


### EDITORIAL DEPARTMENT.

### TO OUR PATRONS—GREETING.

The JOURNAL greets its many friends and supporters, and makes its best bow, in presenting No. 1, of Vol. VIII. This is the "little red-back's" eighth birthday, and it is as proud as a boy with his first red-top boots and knee-breeches. Its pride is commendable. Seven years ago it timidly made its first bow before the Texas profession, a little 6x9 four-form pamphlet,—as an experiment. To-day it is a hundred-page magazine, enlarged and improved in many ways, with a large and growing circulation, a list of *bona fide*, paying subscribers, and a first-class advertising patronage. It has been self-sustaining from the beginning, and is now a paying property. It was stated recently in some medical journal that with the exception of the *Lancet-Clinic*, no journal in America paid its owner a respectable profit. The "Redback" must be excepted, thanks to the warm and steadfast support of the better class of Texas practitioners. It has been fearless and outspoken in the denunciation of "crookedness," both in and out of the ranks, and as staunch in the advocacy of legitimate medicine and a pure profession. It has been entirely independent; and strong in the conviction of the justice of its cause, has dared to maintain it.

Of course it has made some enemies,—enemies of that class who have been made to feel the temper of its blade; but the fact that at the end of the seven years during which it has fought for rational medicine and the Code of Ethics, it is able to thus "spread itself"—to enlarge to six forms, and afford better paper and a larger size—indicates that it has had ten friends for every foe it has made. It is the intention to keep it up to the present standard of excellence, and to cater only to the better class of practitioners. To that end it will continue to be the advocate of a strict conformity to the Code, and to denounce quackery in every form; to lay before its readers that which is best, newest and most practical; to be as far as in its power lies, an aid and help to the busy and hard-working doctor; the exponent of rational medicine in the great rich, growing and prosperous State of

Texas. Its policy will be to give its readers short articles of practical value, rather than long, speculative and theoretical pieces; and we again invite all its readers to make a note of their observations, and send the JOURNAL for publication, any fact or occurrence in their practice which may be of benefit to some embarrassed brother. Many live in sections remote from large libraries, and have not the advantage of consultation, as do those who live in cities; they are thrown on their own resources, and facts observed by one may be of benefit to another, like that recorded in our editorial pages—reported by Dr. Ford—that antipyrine will arrest hemorrhage, for instance. Let us make the JOURNAL the medium of intercommunication between the Texas practitioners of medicine; help us, and we will help you.

In conclusion, we beg to remind our friends that it requires money to conduct a journal; and while we realize that times are hard and money scarce at this season, and for that reason it is not the JOURNAL'S policy to send out bills, this number being the beginning of a new volume the subscription of all our old and many new friends is now due. Some are in arrears. All we ask is to divide with us, and later on, when you sell your yearlings or get in your collections, then you can settle up. With our best wishes for your happiness and prosperity, we present you this souvenir of the JOURNAL'S success.

